# Flux analysis of nutrient substrates in living cells: human glioblastoma cells partially decouple the TCA cycle from oxidative phosphorylation associated with rapid cell proliferation

**DOI:** 10.1186/1753-6561-6-S3-P68

**Published:** 2012-06-01

**Authors:** Lisa Pike, Amy Smift, David Ferrick, Min Wu

**Affiliations:** 1 Seahorse Bioscience, 16 Esquire Road, North Billerica, MA 01862, USA

## 

Cancer cells, unlike normal cells, show selective dependency on certain nutrient substrates. These cells exhibit an addiction to glucose or glutamine, and a lack of flexibility in using a full spectrum of nutrient substrates. This dependency varies widely among different cancer cell types and depends on specific mutations in oncogenes and tumor suppressors, the cellular context in which these mutations occur and epigenetic factors. These wide variations in metabolic states have implications for cancer cell responses to therapy.

To date, methods to analyze cellular substrate metabolism have required either the use of radioactive or stable isotope labeled substrate and/or large quantity of cells, may be labor intensive and have low throughput. Stable isotope labeling coupled with Mass Spectrum and NMR analysis has provided highly detailed information about substrate metabolism, but is relatively inaccessible techniques for a majority of researchers and they require special skills and expertise. We have devised several alternative easier and more rapid experimental methods to analyze substrate flux and metabolic states of cancer cells in a microplate. They have allowed us to analyze substrate flux including glucose, glutamine, and fatty acids, and to interrogate their bioenergetic machineries, glyclolysis and mitochondrial oxidative phosphorylation. These methods measure oxygen consumption rate (OCR) and extracellular acidification rate (ECAR) of living cells, and can determine their dynamic responses to pathway perturbations in real-time allowing substrate flux analysis. Essentially, a large number of cell types (lines) can be analyzed using only small quantities of materials and over a short time periods (1-3 hrs) in a microplate using the XF Extracelllar Flux analyzer.

In proof-of-principle experiments, we investigated substrate flux and the metabolic state of a syngenic pair of human glioblastoma cells, SF188s (slowly dividing) and SF188f (fast dividing) cells. We found that SF188f cells exhibited a substrate shift, away from glycolysis toward glutamine and fatty acid oxidation, in contrast to SF188s cells. The mitochondrial respiratory capacity and basal respiration of SF188f cells were markedly increased, but the ATP production-coupled respiration was greatly reduced. Further, the leaked respiration was also increased compared with the SF188s cells. That is, a large fraction of SF188f cells’ mitochondrial respiration was not devoted to ATP production. We suggest that cancer cells can decouple the TCA cycle from oxidative phosphorylation on an “on demand” basis. This decoupling provides a mechanism by which cancer cells can overcome the metabolic control imposed on the TCA cycle by oxidative phosphorylation, enabling them to meet their extra demand for building block synthesis in addition to energy. This also provides an explanation for the excessive rate of oxygen consumption observed in SF188f cells. In these studies, we also demonstrated that the metabolic state of SF188s and SF188f cells, e.g., glycolysis relative to glutamine oxidation, correlates with their selective susceptibility to inhibitors of glycolysis, 2-deoxyglucose, and glutamine oxidation, aminooxyacetate respectively. Therefore, these methods provide valuable tools for rapid analysis of substrate flux and bioeneregetic machineries in cancer or non-cancer cells. They can also enhance understanding the genetic and epigenetic regulation of cancer metabolism in cancer development.
